# A Model of Interaction between Nicotinamide Adenine Dinucleotide Phosphate (NADPH) Oxidase and Apocynin Analogues by Docking Method

**DOI:** 10.3390/ijms14010807

**Published:** 2013-01-04

**Authors:** Jie Jiang, Hongjun Kang, Xiaoliang Song, Sichao Huang, Sha Li, Jun Xu

**Affiliations:** 1 College of Pharmacy, Jinan University, Guangzhou 510632, China; E-Mails: jiejiang2008@gmail.com (J.J.); hongjunkang@126.com (H.K.); Song1xiaoliang@sina.com (X.S.); sichaohuang.cn@gmail.com (S.H.); 2 College of Medicine, Jinan University, Guangzhou 510632, China

**Keywords:** NADPH oxidase, apocynin analogues, inhibitor, docking

## Abstract

Some apocynin analogues have exhibited outstanding inhibition to NADPH oxidase. In this study, the key interactions between apocynin analogues and NADPH oxidase were analyzed by the docking method. The potential active site was first identified by the SiteID program combining with the key residue CYS378. Afterwards, the compounds in the training set were docked into NADPH oxidase (1K4U) under specific docking constraints to discuss the key interactions between ligands and the receptor. These key interactions were then validated by the consistence between the docking result and the experimental result of the test set. The result reveals that the Pi interaction between apocynin analogues and NADPH oxidase has a direct contribution to inhibition activities, except for H-bond formation and docking score. The key interactions might be valuable to discover and screen apocynin analogues as potent inhibitors of NADPH oxidase.

## 1. Introduction

NADPH oxidase, initially characterized in neutrophils, is a multimeric enzyme consisting of gp91phox (renamed Nox2), p22phox, 3 cytosolic subunits (p47phox, p67phox and p40phox) and the small G-proteins (Rac1/2 and Rap1A) [1,2]. Mutations studies on NADPH oxidase suggest that p47phox requires at least three serines for oxidase activation as follows: a serine at position 379, a phosphorylated serine at position 359 or 370 and a phosphorylated serine at position 303 or 304 [3]. The enzyme plays a key role in immunological host defense for its responsibility to produce reactive oxygen species (ROS). To atherosclerosis, the endothelial NADPH oxidase plays also an important role. By generating superoxidanions, which immediately react with NO to the highly reactive peroxynitrite, the NADPH oxidase is the main source for endothelial dysfunction (an early key event in atherosclerosis) [4–6]. Meanwhile, inhibition of this enzyme represents an attractive therapeutic approach for the treatment of cardiovascular disease, acute lung injury and so on [7,8].

Apocynin (4-hydroxy-3-methoxyacetophenone), originally extracted from the roots of *Picrorhiza kurroa*, has been extensively used for the treatment of diseases associated with chronic inflammation, including jaundice, asthma and cardiovascular diseases [9,10]. Current studies have revealed that apocynin is an efficient non-toxic inhibitor of NADPH oxidase [11]. Therefore, a series of apocynin analogues were synthesized by traditional methods, and their inhibition activities were also tested *in vitro* [12,13]. These works about apocynin analogues have uncovered some potential NADPH oxidase inhibitors better than apocynin and indicated that apocynin would be a valuable lead compound. Some structure-function analyses reveal that the mechanism of apocynin inhibition to NADPH oxidase is a result of peroxidase metabolism, yielding reactive quinones that bind to Cys residues in p47phox and impeding the migration of the cytosolic component of p47phox to the membrane [14–16]. Moreover, mutations of Cys378, which is treated as a conserved residue [17], or one of the other three p47phox cysteines have direct effects in NADPH oxidase activation [18]. The previous studies provide some valuable information that should be constructive to discover and design novel inhibitors of NADPH oxidase. To accelerate the development of apocynin analogues as NADPH oxidase inhibitors, the key interactions between inhibitors and receptor should be analyzed based on the compounds reported.

Compared with traditional methods to explore the interaction between ligands and receptors, the docking method can effectively shorten the cycle time of research and reduce costs. For these reasons, the docking method has been widely used to identify the key interactions between ligands and their receptors [19–22]. The known important interactions would be useful to discover more candidates and guide the synthesis of novel targeted compounds. In this study, the docking method was utilized to explore and validate the key interactions between apocynin analogues and NADPH oxidase combining with our previous experimental result.

## 2. Computational Methods

### 2.1. Database and Software

The structure and inhibitory activities (IC50) of 12 compounds, which served as the training set, were collected from the literature [13]. The test set was composed f 11 apocynin analogues synthesized by our group [12]. The 3D structure of NADPH oxidase (1K4U) was derived from the RCSB protein data bank and consisted of the *C*-terminal SH3 domain of p67phox complexed with the *C*-terminal tail region of p47phox [23]. All the compounds collected in this study would be docked into the 3D structure of 1K4U by the GOLD (Genetic Optimisation for Ligand Docking) program (CCDC Co., Ltd., Cambridge, UK), which is an effective docking program for docking ligands into protein binding sites based on genetic algorithms [24,25].

### 2.2. Preparation of Inhibitors

The structures of ligands have been found to be one of the important determinants for a successful docking. Thus, making pretreatments of inhibitors, such as energy minimization and analysis of the structures, is necessary. First of all, all the 23 ligands’ structures were drawn by sybyl7.0 (Tripos Co., Ltd., St. Louis, MO, USA) and make sure the atoms’ type and chirality is correct. Moreover, the structures’ energy was optimized by the Powell method, and the charges were calculated by the MMFF94 method in the Tripos force field [26–28]. The compounds in the training set were divided into two categories. Relative good inhibitors consisted of the top six inhibition activity compounds, and the other six compounds were treated as relative weak inhibitors.

### 2.3. Identification of the Potential Active Site

The preparation of the receptor and identification of the active site of NADPH oxidase also have a direct contribution to an ideal docking result. Therefore, the 3D structure of NADPH oxidase should be protonated and the water removed primarily. The loop definition, minimization and calculation of charge were then carried out in the Tripos field. After preparation of the NADPH oxidase 3D structure, the SiteID program of Sybyl7.0 was applied to search the potential binding sites on the NADPH oxidase by a flood-fill solvation technique [29]. It has been reported that CYS378 was a key residue in the binding site [14,15,17,18], which would be useful to identify the potential active site according to the results of searching the potential binding site.

### 2.4. Construction and Validation of the Docking Condition

In the premise of the identification of the potential active site with key residue CYS378, the compounds in training set were docked into NADPH oxidase by the GOLD program. In general, an ideal docking result has a close relation with suitable docking constraints, such as the constrained region for the hydrophobic group, ligand flexibility, receptor flexibility, soft potential, H-bond and the docking pose. Hydrogen bond interactions are considered to be key interactions between the N-SH3 domain of p47phox and PRR of p22phox [30,31]. Similar interactions between inhibitors and p47phox may disrupt the association of p47phox-p21phox, which has a direct contribution to NADPH oxidase activity. Therefore, the carbonyl and amino group of the key residue CYS378 of p47phox were treated as a possible H-bone former in the docking process. The other residues in the potential active site were set to be rigid, but compounds in our ligand database were regarded to be flexible, which served as other docking constraints. Compounds with strong inhibition activities to NADPH oxidase usually have stronger interaction with NADPH oxidase compared with the ones with weak inhibition activities. And, docking score is widely used to quantify the interaction between ligand and receptor: a higher score reflects a stronger interaction. Therefore, the docking score should exhibit favorable consistence with inhibition activities of NADPH oxidase. GoldScore has been confirmed to be a high performance docking score [32], so the score function was taken to evaluate the interaction between NADPH oxidase and its inhibitors, as well as inhibition activities in this study.

All the compounds in the training set were docked into the potential active site of NADPH oxidase, identified by SiteID program, under the docking constraints discussed above. After the calculation of the docking score, the docking conformation with the best score was taken as the targeted conformation for each compound in the training set, as well as the test set. At the same time, these compounds were also divided into two groups according to the value of the docking score. The top six docking score compounds were taken as predicted good inhibitors, and the other six compounds were treated as predicted weak inhibitors. As the construction of docking condition was utilized to discover the potent inhibitors of NADPH oxidase, the relative good inhibitors classified by IC_50_ were compared with predicted good inhibitors identified by the docking score to figure out how many predicted good inhibitors would be real good inhibitors. More of these kinds of compounds might show the better prediction ability of this docking method for discovering good inhibitors. For further analysis of the key interaction between NADPH oxidase and its inhibitors, the possible interactions between targeted docking conformation and receptor were discussed for each compound in the training set. Based on the hypothesis of key interaction deduced from the training set, the compounds in the test set were docked into NADPH oxidase to validate the hypothesis. In the validation process, the targeted conformations of compounds in the test set were used to analyze the potential interactions with the receptor. Among these interactions, the correlation between hypothesized key interactions and inhibition activities tested *in vitro* was further explored.

Inhibition activities to NADPH *in vitro* of the compounds in the test set were evaluated by MTT assay in our previous studies [11]. RAW 264.7 cells were seeded into a 96-well microplate at a density of 2.0 × 10^4^ cells/well. The cells were cultured and treated with various doses of compounds (0.1, 1, 10, 100 μM) for 1h prior to the addition of LPS. After 24 h of incubation, MTT was then added (0.5 mg/mL) for 4 h, the medium was removed and the formazan crystals were dissolved in DMSO and isopropanol (1:1). Optical density of the solution in each well was measured at 540 nm. The cell viability rate was calculated as the percentage of MTT absorbance

## 3. Results and Discussion

### 3.1. Training Set and Test Set

**Figure 1:**
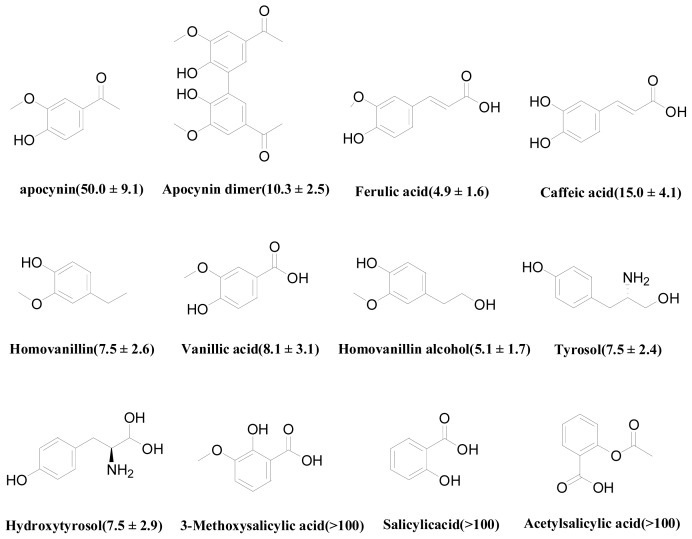
Structures and inhibition activities (IC50: μM) of apocynin analogues as the training set.

Figure 1 illustrates structures and inhibition activities (IC_50_) of the compounds in the training set collected from literature. Analysis of the structures of compounds in the training set reveal that they are all apocynin derivatives and have similar structure fragments. The similarity of structures usually results in a similar interaction or binding style between ligand and receptor. Thus, it is reasonable to believe that these compounds in the training set should bind to the same active site as apocynin. According to the inhibition activities of these compounds in the training set, apocynin dimer, homovanillin alcohol, tyrosol, ferulic acid, hydroxytyrosol and caffeic acid are treated as relative good inhibitors, while others are relative weak inhibitors.

**Figure 2:**
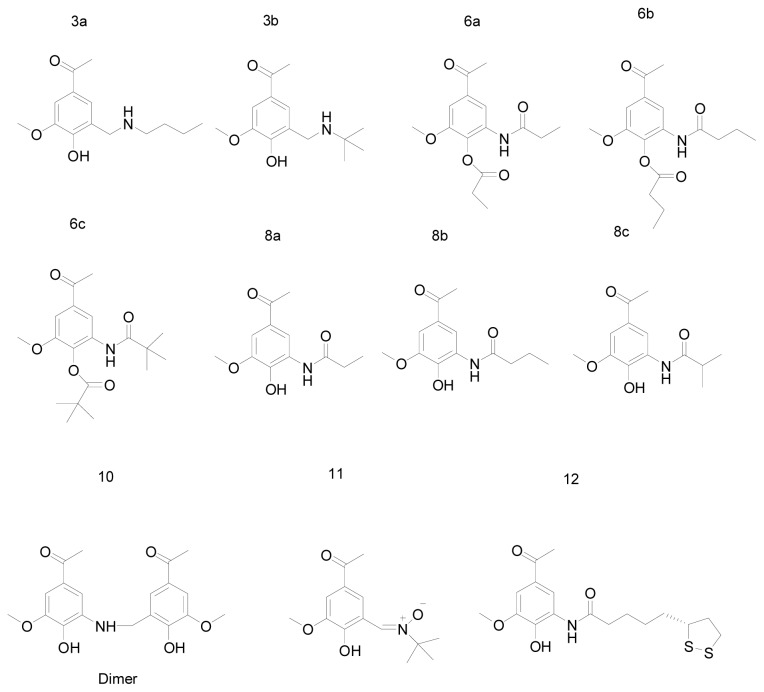
Apocynin analogues synthesized in our group as the test set.

For the potent inhibition activities of some apocynin derivatives, some new analogues were synthesized in our previous work to discover a stronger NADPH oxidase inhibitor (Figure 2). Inhibition activities of these new derivatives to NADPH were also tested *in vitro*, and the result would be valuable to validate the hypothesis concluded from the docking result. In this study, these compounds would be served as a test set, and the consistence between docking result and experimental result was used to validate the interaction model.

### 3.2. Identification of the Potential Active Site

The potential active site is the premise of the docking study about the interaction between NADPH oxidase and its inhibitors. Although some works have been done on the interaction between NADPH and apocynin, the potential active site is not clear, except for some key residues. From the key residues reported, CYS378 is accepted as the key residue to have a close relationship with inhibition activities of inhibitors [14,15,17,18]. Generally, the active site is the pocket formed by a series of conservative amino acid in or on the receptor. Therefore, the SiteID program was used to seek all the possible pockets in or on 1K4U using a flood-fill salvation technique. The Table 1 lists seven pockets identified by SiteID and residues composing the pockets. Considering that CYS378 has been reported as the key residue, the No. 6 pocket containing CYS378 is chosen as the potential active site in this study.

### 3.3. Molecular Docking of the Training Sets

**Figure 3:**
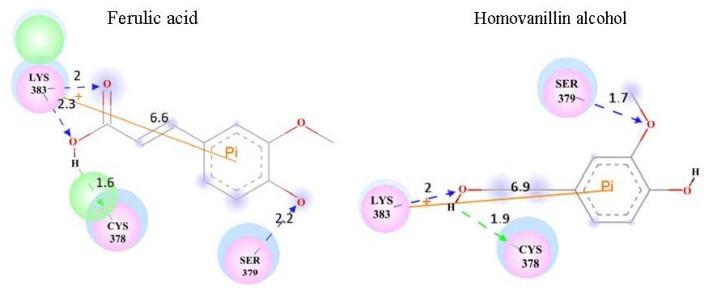

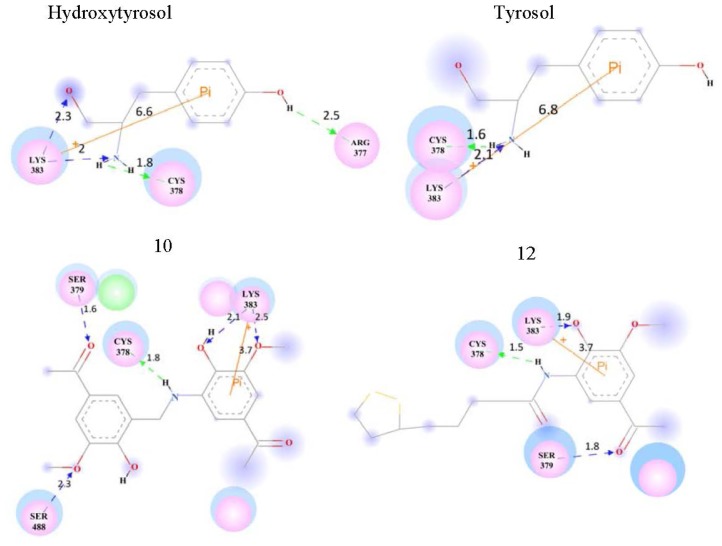
The key interactions between predicted good inhibitors and 1K4U.

After the identification of the potential active site, the compounds in the training set were docked into 1K4U by GOLD, as described in the methods section. In the process, CYS378 treated as H-bond former was the major docking constraint. Docking conformation with the best score was picked out as the preference conformation for each compound. These preference conformations will be taken into subsequent analysis of the key interaction, and their scores are listed in Table 2. In Table 2, relative good inhibitors classified by IC_50_ are emphasized by italics, while the good inhibitors being also predicted as good inhibitors are emphasized in italic and bold type. There are four relative good inhibitors in six predicted good inhibitors, and the remaining compounds also have, to some extent, obvious inhibition activities to NADPH oxidase. The result suggests that the docking score can distinguish the good and weak inhibitors under the specific docking constraints in this study, which also support the importance of H-bond formation between apocynin analogues and the CYS378 of NADPH oxidase. Based on the result, the key interactions between these compounds in the training set and 1K4U were further analyzed, especially for the four good inhibitors supported by both IC_50_ and the docking score. Figure 3 illustrated the potential interactions between predicted good inhibitors and NADPH oxidase. In the figure, H-bonds between ligand and receptor are marked by a dotted arrow, while H-bond acceptors of ligand are colored by blue and green color, indicating the H-bond donor. Beside the H-bonds, Pi interaction between the ligand and the receptor are emphasized by an orange line. The result demonstrates that the interesting Pi interactions between these four compounds and LYS383 of 1K4U are all discovered, but not observed in other predicted good compounds, such as apocynin dimer and caffeic acid. Meanwhile, the Pi interactions were also not found between the predicted weak compounds in the training set and 1K4U, except homovanillin and the SER379 of 1K4U. Furthermore, homovanillin is also identified as a relative good inhibitor according to IC_50_. This result implies that the Pi interaction between apocynin analogues and NADPH oxidase might have a direct contribution to their inhibition activities.

### 3.4. Validation of the Hypothesis

**Figure 4:**
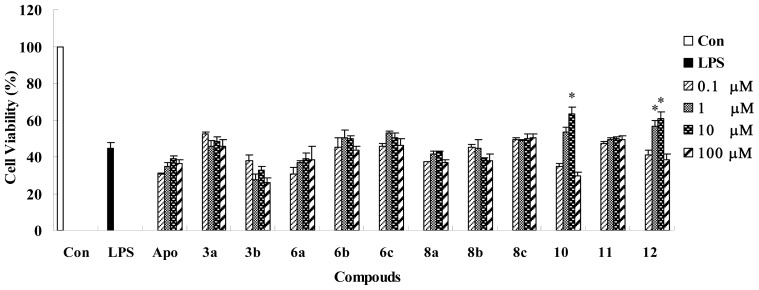
Protective effects against LPS (lipopolysaccharide)-induced cytotoxicity in RAW 264.7 macrophage cells [12].

Based on the docking result and analysis of the interaction, a hypothesis was put forward to screen and discover apocynin analogues as potent inhibitors to NADPH oxidase. In the hypothesis, the better inhibition activity is supported by Pi interaction, as well as the better docking score and H-bond formation with CYS378. To validate the hypothesis concluded from docking study between the training set and 1K4U, the compounds in the test set were docked into 1K4U by the GOLD program under the same docking constraints. The docking scores were calculated and listed in Table 2. Moreover, the interactions between these compounds and 1K4U were analyzed to find out which compounds have Pi interaction and H-bond formation with the receptor, except for the better docking score. The result reveals that No. 10 and 12 have a Pi interaction with LYS383 of 1K4U, just as the four compounds in the training set (Figure 3). Simultaneously, the two compounds also show a top 1 and top 3 docking score in the test set and H-bond formation with CYS378. Thus, No. 10 and 12 are chosen as potent NADPH oxidase inhibitors according to the hypothesis concluded above. It is encouraging that the predicted result is validated by our previous work about the inhibition activities of compounds in the test set (Figure 4) [12]. Inhibition to NADPH oxidase can effectively protect against LPS (lipopolysaccharide)-induced cytotoxicity in RAW 264.7 macrophage cells and promote cell viability. Therefore, better cell viability might suggest better inhibition to NADPH oxidase. As shown in Figure 4, the result reveals that No. 10 and 12 compounds have obvious better inhibition activities to NADPH oxidase *in vitro* compared with apocynin and other compounds in the test set. The consistency between predicted and experimental results demonstrates the hypothesis about the importance of Pi interaction. Meanwhile, the selection of potent inhibitors of NADPH oxidase should not only depend on H-bond formation with CYS378 and the docking score, but also the Pi interaction.

## 4. Conclusions

In this paper, a model of interaction between NADPH oxidase and apocynin analogues was constructed by the docking method. A potential active site was identified by the SiteID program and the key residue reported from previous studies. Based on the active site, the compounds in the training set were docked into NADPH oxidase (1K4U) following the specific docking constraints by GOLD. The docking result reveals that the docking score can well distinguish the good and weak inhibitors, while the further analysis of interaction between apocynin analogues in the training set and 1K4U demonstrates that Pi interaction might have a direct contribution to inhibition activities, except for H-bond formation and the docking score. And, the hypothesis was supported by the consistency between the docking result of the test set and our previous experimental test *in vitro*. Therefore, it is reasonable to believe that the interaction model should be helpful to discover and screen apocynin analogues as potent inhibitors of NADPH oxidase.

## Figures and Tables

**Table 1:** The pockets in or on 1K4U identified by SiteID.

Pocket	Residues
1	GLN461, VAL462, VAL486, LYS489, GLU492, TRP494, LEU495, LYS508, ASP531, SER460, THR516
2	GLN461, VAL462, GLU463, ILE483, ILE484, LEU485, LYS458, SER514, SER460, ALA515
3	PRO363, ALA364, PRO366, PRO367, ARG368, PRO369, ASN491, GLU492, GLU493, TRP494, LYS508
4	GLN362, PRO363, ALA364, VAL365, PRO366, ARG368, SER467, TYR468, GLU469, GLN472, ASP475, TRP494, PHE510
5	LYS385, ALA470, GLN472, PRO473, GLU474, ASP475, LEU476, GLU477, PHE478, GLN479, ASP482, ILE484, GLY497, SER499, LYS500, LYS502, VAL503, GLY504
6	ARG377, CYS378, SER379, LEU487, SER488, LYS489, GLU496
7	ARG368, PRO369, ALA371, ILE374, LYS385, LEU386, SER388, GLU474, ILE505

**Table 2:** Scoring results of the molecular docking training set.

Compound			NADPH oxidation	GOLD Score	Pi interaction

			IC_50_^a^(μm)(Experimental)		
Training set	Predicted good	Apocynin dimer	10.3 ± 2.5	45.0082	-
		**Homovanillin alcohol**	**5.1 ± 1.7**	**35.1074**	**LYS383**
		**Tyrosol**	**7.5 ± 2.4**	**32.8184**	**LYS383**
		**Ferulic acid**	**4.9 ± 1.6**	**31.7482**	**LYS383**
		**Hydroxytyrosol**	**7.5 ± 2.9**	**28.9217**	**LYS383**
		Caffeic acid	15.0 ± 4.1	27.8341	-

	Predicted weak	3-Methoxysalicylic acid	>100	27.3678	-
		Apocynin	50.0 ± 9.1	27.0364	-
		Vanillic acid	8.1 ± 3.1	26.6326	-
		Homovanillin	7.5 ± 2.6	26.0688	SER379
		Acetylsalicylic acid	>100	25.7012	-
		Salicylic acid	>100	23.2333	-

Test set		**12**	**-**	**52.0590**	**LYS383**
		6b	-	42.8667	-
		**10**	**-**	**41.1901**	**LYS383**
		6c	-	40.7741	-
		8c	-	40.6892	-
		3b	-	38.6765	-
		8a	-	38.2455	-
		8b	-	37.7346	-
		11	-	37.4821	-
		6a	-	36.7572	-
		3a	-	36.4118	-

a Concentration (μm) of inhibitors in training set required to achieve 50% inhibition of NADPH oxidase activity.
